# Information Entropy Theory Applied to the Dip-Phenomenon Analysis in Open Channel Flows

**DOI:** 10.3390/e21060554

**Published:** 2019-06-01

**Authors:** Domenica Mirauda, Maria Grazia Russo

**Affiliations:** 1 School of Engineering, Basilicata University, Viale dell’Ateneo Lucano 10, 85100 Potenza, Italy; 2 Department of Mathematics, Computer Science and Economics, Basilicata University, Viale dell’Ateneo Lucano 10, 85100 Potenza, Italy

**Keywords:** Shannon’s information entropy, velocity-dip-position, open channel flows, maximum velocity, mean velocity, theoretical model

## Abstract

The knowledge of the fluid discharge in free surface flows requires a great number of velocity measurements along the whole cross-section, taking up a large amount of time, using expensive equipment, and employing specialized labor. To overcome these obstacles, various models have been developed thus far that show how to estimate the mean velocity through the maximum velocity. In three-dimensional open channels, the maximum velocity can be located below the free surface because of the presence of secondary flows mainly originating by the sidewalls, an occurrence known as dip-phenomenon. In this condition, predicting the maximum velocity position is quite difficult and has always represented a challenge to most hydraulic engineers and researchers. In the present study, a mathematical model derived from the information entropy theory is proposed to evaluate the velocity-dip-position over the entire cross-section of both wide and narrow open channels, thus overcoming the limitations of the existing methods. Large literature measurement sets, collected in uniform and non-uniform flows, were used to test the validity of the model, showing good agreement with the experimental data and providing an accurate estimation of the dip-position.

## 1. Introduction

The water discharge of an open channel flow, an essential element for the effective management of water resources, is estimated through the measurement of the cross-section mean velocity. An accurate evaluation of the latter depends on the availability of a great number of velocities sampled in all portions of the flow area, which requires considerable manpower, time, and costs, in addition to also being dangerous during high floods. In the last decades, studies on the information entropy theory [1,2,3] have showed that the mean velocity can also be predicted from the value of the maximum velocity, thus reducing the amount of velocity measurements and making the calculation of the water discharge easier. However, in the case of open channels, the location of the maximum velocity is not always known a priori because it can vary according to the features of the flow and the shape of the cross-section.

For more than a century, scientists [4,5,6,7,8] have highlighted the occurrence of the maximum velocity below the free surface for most open channels, known as velocity-dip-phenomenon. They have also found a different location of the maximum velocity along the cross-section going from narrow to wide open channels. In particular, for narrow channels, when the aspect ratio, *A_r_*, is low (e.g., lower than 5 for [9]), the maximum velocity generally occurs in the cross-section central portion, where the secondary currents direct the flow with relatively high streamwise momentum, causing a marked influence on the axial velocity. For wide channels, the maximum velocity is usually found in the sidewall regions instead, even though different experiments, such as some by [10], have also shown its presence in the central section due to the variation of the bed roughness or bed elevation along the lateral direction.

To predict the dip-position in open channels, different numerical, empirical, and analytical models have been developed in the literature ([11,12,13,14,15,16,17,18,19,20,21,22,23] to name a few).

In 2000, Sarma et al. [11] proposed a binary model combining a log law in the inner region and a parabolic law in the outer region in smooth and rough open channels, finding a relationship between its junction point and the maximum velocity location. In fact, it is equal to 0.5 *D* (where *D* is the water depth) when there is no dip-phenomenon and decreases from 0.5 *D* to 0 when the dip-phenomenon occurs.

A study by [12] found a link between the dip-phenomenon and the aspect ratio. In detail, for narrow channels, the maximum velocity is located in the central portion of the channel and below the free surface, while for wider channels it moves towards the sidewall region and near the free surface.

In 2002, Chiu and Tung [13] derived an empirical model which related the location of the maximum velocity from the free surface to the velocity distribution parameter using the information entropy concept.

In smooth uniform open channel flows, Yang et al. [14] described the position of the cross-section maximum velocity through a dip-modified log law, able to reconstruct the velocity profile from the bed to just below the free surface, and transversely from the central line to the sidewalls. With increasing channel width, the dip-phenomenon disappeared in the central portion and was detected near the sidewall region.

Using a modified log law, Wang and Cheng [10,15] calculated the velocity-dip-position numerically, applying the zero turbulent shear stress condition to the maximum velocity location. Their method was validated on flumes with secondary currents created by artificial bed strips.

In 2008, Bonakdari et al. [16] developed a sigmoid model to detect the velocity-dip-position in the central cross-section of both smooth wide and narrow open channel flows. However, it did not satisfy the asymptotic boundary conditions in which the maximum velocity occurred between the lowest value of 0.5*D* for *A_r_*→ 0 and the highest value of 1*D* for *A_r_*→∞.

Through laboratory and field measurements, Guo and Julien [17] followed an empirical procedure to determine the dip-position from the bed, starting from the modified log-wake law and extending its use to turbulent open channel flows.

In order to predict dip-phenomena and accurately reproduce the velocity profiles in flow regimes with rough walls, Absi introduced a simple dip-modified-log-wake law in 2009 [18] and a full dip-modified-log-wake law in 2011 [19], both based on the Reynolds-Averaged Navier–Stokes equations and on a log-wake modified eddy viscosity distribution.

Later, Guo [20] integrated the existing empirical knowledge into Guo and Julien’s [17] modified log-wake law for smooth rectangular open channel flows, highlighting that the dip-position shifted from the free surface to half the flow depth exponentially as the aspect ratio decreased from infinity for wide channels to zero for pipe flows.

In the same year, Pu proposed a velocity distribution law derived directly from the Navier–Stokes momentum equation, validated for both wide and narrow open channel flows with rough and smooth beds, obtaining a dip-velocity law similar to the one of [21] which, differently from the latter, satisfied both the asymptotic boundary conditions.

Recently, referring to the information entropy theory, Kundu [22] obtained a relationship between the velocity-dip-position and the aspect ratio, without the need of knowing the velocity distribution. The model, even if deriving from a theoretical approach and, thus, valid for different flow conditions, is only directed to the central section of an open channel and for a low range of aspect ratios (*A_r_* < 12).

In 2018, Mirauda et al. [23] introduced an analytical method to predict the velocity dip-position based on the assumption that the velocity entropy is equal to the entropy of the maximum velocity position from the bed non-dimensionalised against the flow depth, and the obtained results were tested on some field data.

This paper proposes a theoretical model, stemming from the principle of the maximum informational entropy, to estimate the velocity-dip-position in open channels, knowing only one single entropic parameter, *M*. The latter seemed to keep constant over the entire cross-section despite the varying water discharge in [24,25,26,27] and over the entire reach for rivers with the same morphological characteristics in [28]. Recently, Moramarco and Termini [29,30] analyzed the constancy of *M* both in a straight laboratory channel for different roughness boundaries and in a meandering flume. In [29], the same authors found the value of the entropic parameter equal both for smooth/rough sidewalls and rough bed, as well as for bottom vegetation at high flow submergences and lower stem concentrations. In [30], instead, a variation of *M*, accentuated in the absence of bed deformation, was observed along the bend, in agreement with previous results by [24]. The here-proposed model was validated through a detailed error analysis in a wide range of experimental data sets and the results support its application both in the central line and over the entire cross-section of wide and narrow open channels having different bed and sidewall roughness conditions, regular and irregular geometric shapes, and various water discharges and flow depths. Therefore, since the model does not depend on specific kinematic or dynamic flow characteristics, it carries fewer limitations compared with most of the methods so far developed in the literature.

## 2. Theoretical Background

The model here developed finds its theoretical basis in Shannon’s information entropy [31] of the dimensionless dip-position, *ξ_d_*, defined as [22]

(1) $$H\left(\xi_{d}\right)=\int_{\xi_{*}}^{D_{*}}f\left(\xi_{d}\right)ln[f\left(\xi_{d}\right)]d\left(\xi_{d}\right),$$

where $\xi_{*}$ and $D_{*}$ are the lower and upper bounds of *ξ_d_*, respectively, and *f*(*ξ_d_*) is the probability density function so that *f*(*ξ_d_*) is the probability of *ξ_d_* being between $\xi_{*}$ and $D_{*}$. To obtain the least biased probability of *ξ_d_*, the entropy function H is maximized according to [32,33,34] and subjected to some specific constraints. The first constraint is expressed by

(2) $$\int_{\xi_{*}}^{D_{*}}f\left(\xi_{d}\right)d\xi_{d}=1,$$

while the second constraint is given as the mean of *ξ_d_*

(3) $$\int_{\xi_{*}}^{D_{*}}\xi_{d}f\left(\xi_{d}\right)d\xi_{d}=\bar{\xi_{d}}.$$

The maximization function *H*(*ξ_d_*) uses the Lagrange multipliers *λ*_0_ and *λ*_1_, ignoring the integration signs as follows:

(4) $$L_{0}=-f\left(\xi_{d}\right)ln\left[f\left(\xi_{d}\right)\right]+\lambda_{0}f\left(\xi_{d}\right)+\lambda_{1}\xi_{d}f\left(\xi_{d}\right).$$

Differentiating the Lagrangean function *L* with respect to f and setting it equal to zero, one gets

(5) $$\frac{\partial L_{0}}{\partial f}=-ln\left[f\left(\xi_{d}\right)\right]-1+\lambda_{0}+\lambda_{1}\xi_{d},$$

from which the probability density function, *f*(*ξ_d_*), including the Lagrange multipliers, is obtained:

(6) $$f\left(\xi_{d}\right)=exp\left(\lambda_{0}-1\right)exp\left(\lambda_{1}\xi_{d}\right).$$

Inserting Equation (6) in Equation (1), the entropy function *H*(*ξ_d_*) is determined:

(7) $$H\left(\xi_{d}\right)=\frac{exp\left(\lambda_{0}-1\right)}{\lambda_{1}}\left[exp\left(\lambda_{1}D_{*}\right)\phi \left(D_{*}\right)-exp\left(\lambda_{1}\xi_{*}\right)\phi \left(\xi_{*}\right)\right],$$

where Φ($D_{*}$) and Φ($\xi_{*}$) are equal to

(8) $$\phi \left(D_{*}\right)=2-\lambda_{0}-\lambda_{1}D_{*},$$

(9) $$\phi \left(\xi_{*}\right)=2-\lambda_{0}-\lambda_{1}\xi_{*}.$$

The two Lagrange multipliers, *λ*_0_ and *λ*_1_, can be obtained by substituting Equation (6) in Equations (2) and (3):

(10) $$\frac{exp\left(\lambda_{0}-1\right)}{\lambda_{1}}\left[exp\left(\lambda_{1}D_{*}\right)-exp\left(\lambda_{1}\xi_{*}\right)\right]=1,$$

(11) $$f\frac{exp\left(\lambda_{0}-1\right)}{\lambda_{1}}\left[exp\left(\lambda_{1}D_{*}\right)\left(\lambda_{1}D_{*}-1\right)-exp\left(\lambda_{1}\xi_{*}\right)\left(1-\lambda_{1}\xi_{*}\right)\right]=\bar{\xi_{d}}.$$

In more detail, *λ*_0_ and *λ*_1_ have been determined, requiring the knowledge of the asymptotic boundary conditions by the two non-linear Equations (10) and (11), numerically solved through the @MATLAB function “fsolve” which implements a trust-region method for minimization with the Dogleg implementation. The values of $\xi_{*}$ and $D_{*}$ have been set to 0.5 and 1, respectively, according to literature studies by [20,35].

## 3. Experimental Data

Table 1 reports the large set of experiments related to laboratory flows, having either fixed [25,27,36,37] or mobile [38,39,40,41,42,43] bed, and to field measurements in both high and low water discharges [44,45,46].

The velocities of [36] were acquired in uniform, turbulent (i.e., with Reynolds numbers ranging from 1.6 × 10^5^ to 5.6 × 10^5^), and subcritical (i.e., with Froude numbers from 0.23 to 0.48) laboratory flows, where two types of roughness (rough plate and gravel bed) were applied. The first type was made up of a single layer of crushed grains glued to the channel bottom with a height equal to 0.0048 m, while the second one was created by a quasi-uniform gravel covering the original floor with a thickness of about 0.10 m. The low values of the aspect ratio, *A_r_* < 7 (narrow channels), highlighted the influence of secondary currents on the velocity profile shape.

The experiments of [37] occurred in a glass-walled flume (9.0 m long, 0.6 m wide, and 0.6 m deep) with rough bottom represented by a plastic doormat 10 mm thick, where the point velocities—taken for various subcritical (0.14 ≤ *Fr* ≤ 0.76), turbulent (3.4 × 10^4^ ≤ *Re* ≤ 1.7 × 10^5^), and uniform flows—showed a progressive disappearance of sidewall effects, going from narrow (*A_r_* = 4.2) to wide (*A_r_* = 12.0) channels.

The researchers in [25,27] carried out tests on a tilting flume (9 m in length and with a cross-section of 0.5 × 0.5 m) with a set of wooden spheres (*d* = 0.035 m) placed on the bottom, in order to simulate the behavior of a natural channel with homogenous roughness. The measurements were conducted in steady flow conditions for low values of relative submergence ranging from 2 to 7 and, for *A_r_* < 7, when the maximum velocity generally occurred below the free surface.

A study by [38] evaluated the streamwise velocity distribution in a laboratory steel channel (0.3 × 0.36 × 12 m), both with clear water and with sediment-laden flows. The experiments were performed varying the slope (0.0185 ≤ *i* ≤ 0.025), discharge (0.074 ≤ *Q* ≤ 0.085 m^3^/s), and depth (0.11 ≤ *D* ≤ 0.15 m) and using three different sand sizes (coarse *d*_50_ = 1.3 mm, medium *d*_50_ = 0.94 mm, and fine *d*_50_ = 0.275).

Data were collected by [39] in two recirculating flumes, the first one of plywood (2.4 × 0.6 × 46 m) and the second one with clear-plastic sidewalls and stainless-steel floor (0.6 × 0.8 × 18 m), having various sand sizes (0.19, 0.27, 0.28, 0.32, 0.33U, 0.33G, 0.45, 0.47, 0.54, and 0.93 mm) coming from rivers, in order to nearly duplicate the flow conditions in alluvial channels. After making a given discharge of water-sediment mixture recirculate at a preselected slope until reaching the equilibrium conditions, the measures were detected and then employed to determine the effects of bed material and suspended sediment on the velocity profiles.

The tests by [40] were performed in a tilting laboratory channel (13 m long and 0.267 m wide) on turbulent, subcritical, and uniform flows both in clear water and with suspended sediment, adding well-sorted natural sands of different diameter (*d*_50_ = 0.15 mm, 0.19 mm, and 0.24 mm). In both saturated (in equilibrium with a flat sand bed) and unsaturated (starved-bed with no sand bed) flows, Lyn showed how the shape of the velocity-defect profiles was similar to the clear water case, except for the near-bed region, due to the different sand sizes used.

A study by [41] measured a large amount of velocity profiles along the axis of a recirculating flume (0.356 m wide × 15 m long), adjusting the slope to maintain the flow uniform and the fluid discharge (0.064 m^3^/s), average flow depth (0.169 m), and energy gradient constant (0.002). Coleman monitored changes in the velocity distribution resulting from systematic increases in suspended sediment concentration (three different sands of nominal diameters equal to 0.105 m, 0.210 m, and 0.420 mm).

Similarly to Coleman’s, Valiani’s experiments [42] were performed in a 0.37 m wide × 10.5 m long Plexiglas flume for fixed flow rate (0.022–0.024 m^3^/s), slope (0.002), and depth (0.10 m) and for changing grain sizes (0.150 mm, 0.106 mm, and 0.075 mm), gradually increasing the solid discharge until saturation of the uniform flow transport capacity. The tests highlighted that, at different cross-sections, the maximum velocity generally occurred below the free surface.

A study by [43] monitored the variation of instantaneous longitudinal and vertical velocities at the central line of a laboratory channel cross-section (0.6 m wide) by adding an amount of sand in several steps to a clear water flow in a uniform condition until the presence of a thin layer of sediments was observed on the bed. The experiments have been performed using sand particles of diameter *d*_50_ = 0.135 mm and *d*_50_ = 0.230 mm, in conditions of subcritical (0.63 ≤ *Fr* ≤ 0.85) and turbulent (2.33 × 10^5^ ≤ *Re* ≤ 3.14 × 10^5^) flows and with values of aspect ratio large enough (*A_r_* ≥ 5) to predict a bi-dimensional flow.

Moramarco et al. [44] developed an expeditive methodology to detect the water discharge in open channels, using velocity data collected over a period of 20 years in four sections of the upper Tiber basin (Central Italy), equipped with a remote ultrasonic water level gauge. The cross-sections investigated belong to reaches with characteristics similar to those of alluvial meandering channels with broad, well-defined floodplains, a slight entrenchment, low slopes, and a riffle-pool bed morphology.

Field data were acquired by [45] at three ungauged sections of the river Alzette (Grand Duchy of Luxembourg) with either bridge piers or concrete walls on their banks and beds of gravel or sand/silt, and at three gauged sections of the main Basilicata rivers (southern Italy) with coarse or fine sediment on the bottom and anthropized or natural reaches. The results showed the influence of the channel pattern (straight, meandering, or braided), the bed material (fine sand, silt, or gravel) and the reach typology (regularised by reinforced concrete or naturally covered by vegetation) on the velocity distributions.

Sets of velocity measurements were gathered by [46] on several monitoring cross-sections along different streams in southern Italy (the rivers Follone and Amato in Calabria and the Basento, Sinni, Agri, and Cavone in Basilicata), considering various slopes (0.1–1%), mean bed sediment diameters (*d*_50_ = 3–8.6 cm), high (4 ≤ *D*/*d*_50_ ≤ 17) and low (1.2 ≤ *D*/*d*_50_ ≤ 4) relative submergences, both in in-bank (0.15 ≤ *Q* ≤ 9 m^3^/s) and low-stage (0.017 ≤ *Q* ≤ 1.9 m^3^/s) flow conditions.

**Figure 1:**
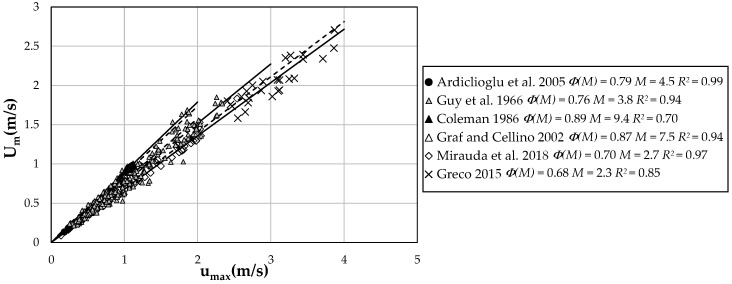
Relation between mean and maximum cross-section velocity.

Figure 1 shows the dependence between *U_m_* and *u_max_* for some experiments, reported in Table 1. The angular coefficient of the best-fit line relative to the observed values of velocities represents the dimensionless function Φ(*M*), which is linked to the entropic parameter, *M*, through the following law [1,2]:

(12) $$\Phi \left(M\right)=\frac{U_{m}}{u_{max}}=\frac{e^{M}}{e^{M}-1}-\frac{1}{M}\;.$$

The high value of the correlation coefficient, *R*^2^, obtained for all the curves of Figure 1 highlights how Φ*(M)* and *M* can be assumed constant for a same cross-section with varying water discharge.

The constancy of *M* for each data set is a sign of the channel section tendency to establish and maintain an equilibrium state under a wide range of flow conditions to which a single value of velocity entropy corresponds, according to the following formula [13]:

(13) $$H\left(\frac{u}{u_{max}}\right)=\int_{0}^{1}f\left(\frac{u}{u_{max}}\right)lnf\left(\frac{u}{u_{max}}\right)d\left(\frac{u}{u_{max}}\right)=1+\ln\frac{e^{M}-1}{M}-\frac{Me^{M}}{e^{M}-1}.$$

In particular, Chiu and Said [3] demonstrated how an erodible channel reaches an equilibrium state, having a single value of *M* and *H(u/u_max_)*, adjusting its hydraulic and geometric characteristics (cross-section, slope, roughness, alignment, velocity distribution, and, perhaps, sediment transport). On the other hand, a non-erodible channel can only adjust its water depth and pattern of velocity distribution to maintain its equilibrium state and the relative *M* and *H(u/u_max_)*. Consequently, non-erodible channel sections are more capable of adapting their velocity distribution to the changing discharge in a wider range of possible *M* and *H(u/u_max_)* values than the erodible channel sections [47]. Furthermore, a comparison of different rivers has underlined that their respective cross-sections, having the same morphological characteristics, might have the same value of *M* [28].

As observed in Figure 1, the value of *M* is between 2.3 and 9.4, similar to [48], thus demonstrating the basic role played by section geometry in determining the location of maximum and mean velocity, and, hence, the velocity distribution entropy. In detail, higher values were found for laboratory data compared with field data, which highlights how rivers are able to reach a greater velocity entropy, and so reduce the *M* value, thanks to the increasing erosive sediment concentration in the flow. Aside from this, these rivers often show the maximum velocity very close to the free surface, having higher values of aspect ratios. Laboratory channels are instead characterized by a greater value of *M* and thus minor velocity entropy, having a more uniform velocity distribution and a more accentuated velocity-dip. Therefore, the section geometry plays an important role in determining both the value of the velocity entropy and the location of the maximum velocity.

**Figure 2:**
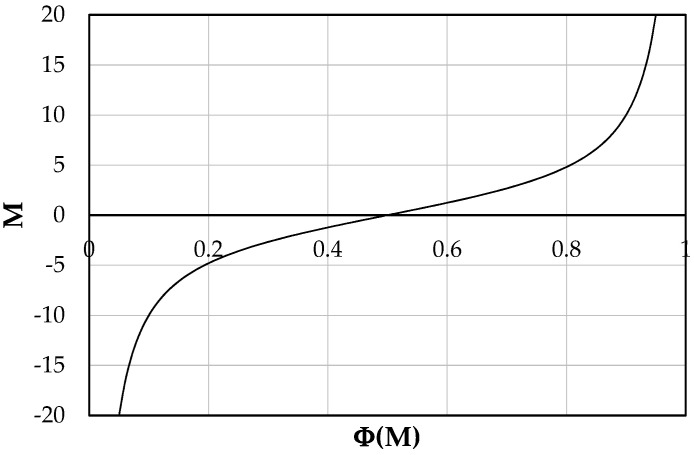
Trend of *M* against Φ*(M)*.

In addition, flume data show a wide range of the entropic parameter compared with the ones detected in natural channels, probably due to the trend of *M* against Φ*(M)*, described by Figure 2. Therefore, a wide range of both parameters are observed for low *M*, while for its higher values a slight variation of the coefficient Φ*(M)* is noted.

## 4. Proposed Model

**Figure 3:**
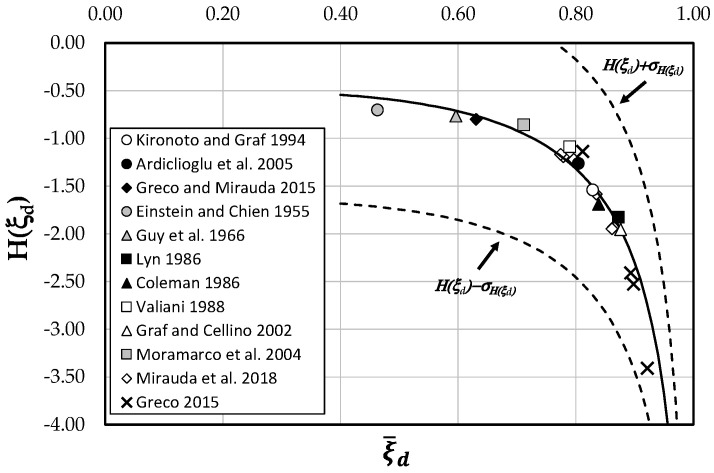
Analytical law for the entropy *H*(*ξ_d_*) in function of ${\bar{\xi }}_{d}$.

By plotting the data of *H*(*ξ_d_*) against the observed means of *ξ_d_*, it is possible to recognize a logarithmic behavior (Figure 3). In order to find an analytical model, a nonlinear least square approximation scheme was used. In particular, the @MATLAB function fit (using the Levenberg–Marquardt approach) gave

(14) $$H\left(\xi_{d}\right)=-a+bln{\left(c\left(1-{\bar{\xi }}_{d}\right)\right)}^{2},$$

where the values of the experimental coefficients *a*, *b*, and *c* are equal to 0.5, −0.4, and 1.2, respectively.

Equation (14) allows simplifying the analytical treatment described in the previous paragraph.

As one can see, most of the data are rather close to the theoretical curve, since 100% of them fall within the 95% confidence interval. Therefore, the law agrees well with the experimental measurements over a wide range of channels.

**Figure 4:**
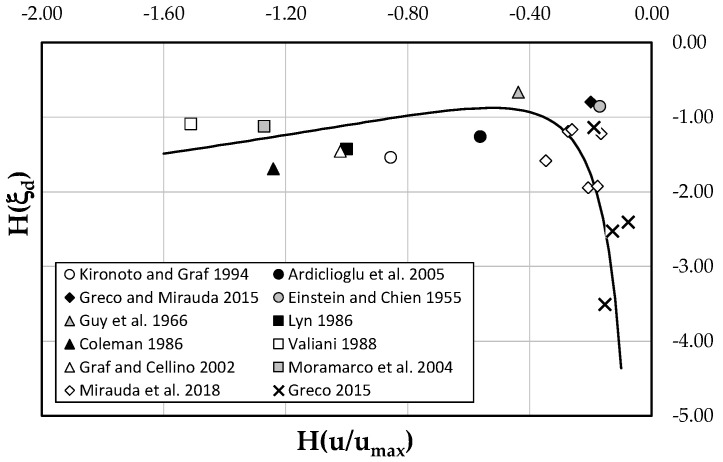
Validation of the proposed model.

To establish the relation between the entropy of *ξ_d_* and the flow domain, a theoretical model needs to be formulated using a nonlinear least square approximation scheme (@MATLAB function fit with the Levenberg–Marquardt approach). The computed values of *H*(*ξ_d_*) from Equation (14) in function of the velocity entropy *H(u/u_max_)*, estimated through Equation (13), are reported in Figure 4. According to the graph, it is clear that for small values of *H(u/u_max_)*, the function *H*(*ξ_d_*) has a logarithmic behaviour, while for higher values of *H(u/u_max_)* the velocity entropy shows an exponential decay. Therefore, the *H(ξ_d_)* model in the flow domain was derived in the form of:

(15) $$H\left(\xi_{d}\right)=\frac{\alpha }{exp^{H\left(\frac{u}{u_{max}}\right)}-1}-\beta ln\left|H\left(\frac{u}{u_{max}}\right)\right|,$$

where the values of *α* and *β* were obtained as 0.7 and 1.3, respectively, after fitting Equation (15) with the experimental data of Table 1. Figure 4 underlines, for the field data, a higher velocity entropy asymptotically tending to zero and a wide range of lower dip-position entropies. In laboratory channels, instead, a large range of lower velocity entropies and high *H*(*ξ_d_*) close to a constant value are mostly observed. The different behavior of the channels with rigid boundaries is explained from the fact that, in order to maintain an equilibrium state under various flow conditions, they are capable of adjusting the velocity distribution by modifying the maximum velocity and the dip-position in a wider range of velocity entropies, as mentioned in the previous paragraph.

Substituting Equation (14) in Equation (15), reformulating and solving it for *ξ_d_*, the following is obtained:

(16) $$\xi_{d}=1-\frac{D}{{\left|H\left(\frac{u}{u_{max}}\right)\right|}^{B}}\left[exp\left(\frac{A}{exp\left(H\left(\frac{u}{u_{max}}\right)\right)-1}\right)\right],$$

where *A* = α/2b, *B* = β/2b, and $D=exp^{a/2b}/c$.

The velocity-dip-position can thus be calculated from Equation (16) within the open channel cross-sections with any given entropic parameter, *M*.

**Figure 5:**
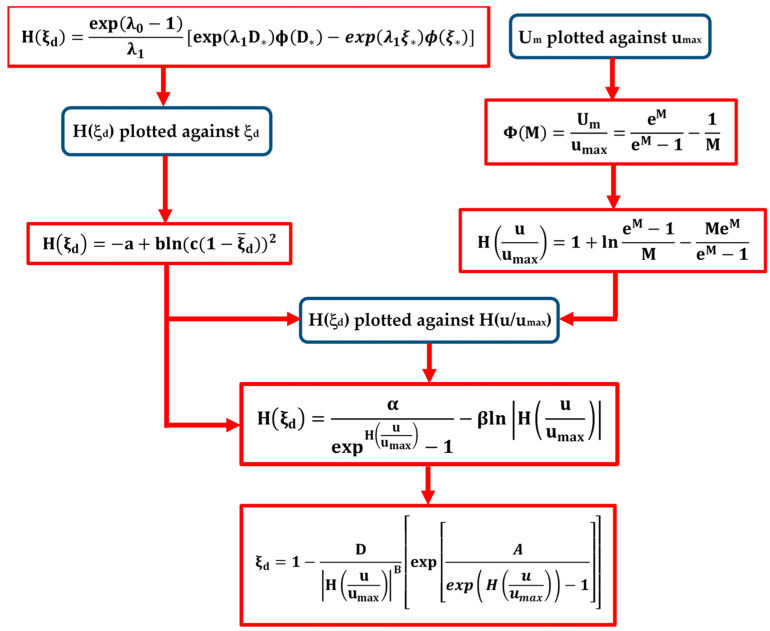
Schematic representation of dip-position estimation procedure.

All the steps of the procedure used to obtain Equation (16) are listed below and summarized in the flow chart of Figure 5:

1. the observed pairs (*U_m_, u_max_*) of the literature experiments were plotted in Table 1 in order to evaluate the coefficient Φ*(M)* for each data set;
2. the entropic parameter *M* was calculated from Equation (12) once Φ*(M)* was known;
3. the velocity entropies *H(u/u_max_)* were estimated from Equation (13);
4. the values of *H(ξ_d_)* were evaluated from Equation (7) for each data set and plotted against the mean values of *ξ_d_* to obtain Equation (14);
5. the values of *H*(*ξ_d_*) were recalculated from Equation (14) and plotted against the values of *H(u/u_max_)* to extract Equation (15);
6. Equation (16) was determined by substituting Equation (14) in Equation (15).

For a new set of data, once the parameter *M* is estimated through the pairs (*U_m_*, *u_max_*) using the linear entropic relation of Equation (12), and thus giving the corresponding velocity entropy *H(u/u_max_)*, the value of the dip-position can be evaluated by applying only Equation (16).

## 5. Discussion of Results

Equation (16) can evaluate the dip-position whenever the entropic parameter, *M*—which represents the characteristics of an open channel flow—is known, and thus can estimate the mean velocity at any time. In particular, once the maximum velocity in a given cross-section has been measured, and exploiting the advantage of *M* being a constant, the mean flow velocity can be calculated easily from Equation (12).

The reliability of the theoretical model here developed is confirmed by the experimental coefficients obtained from a wide set of data referring to channels with various characteristics, such as alignment, slope, roughness, geometric shape, and hence velocity and shear stress distributions.

**Figure 6:**
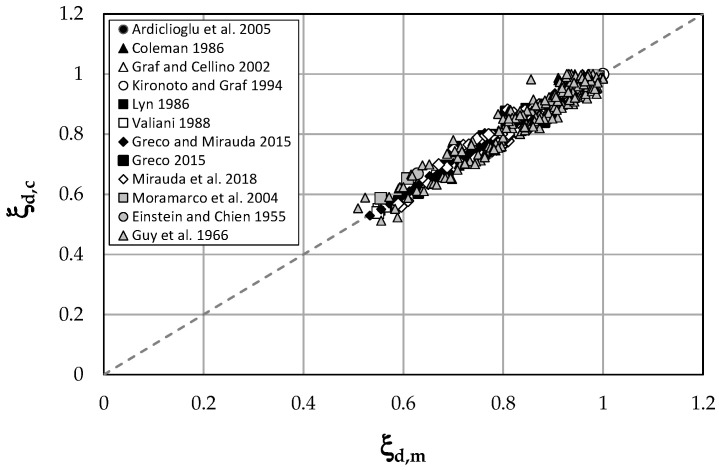
Theoretical and measured values of dimensionless dip-positions for all investigated data.

A further confirmation of the method accuracy is given by the comparison between the dimensionless dip-positions predicted by Equation (15) and the ones measured in all the investigated data for each value of water discharge and flow depth (Table 1), reported in Figure 6.

The high value of the correlation coefficient, *R*^2^ = 0.94, shows how the proposed information entropy-based model agrees well with the experimental data over a large range of investigated open channel flows.

In addition, the model performance is supported by a detailed error analysis based on four different statistical parameters:

$$APRE=\frac{1}{n}\overset{n}{\underset{i=1}{\sum }}\frac{\left|\xi_{d,c}-\xi_{d,m}\right|}{\xi_{d,m}}\times 100\left(\%\right),$$

$$SSRE=\sum_{i=1}^{n}\frac{{\left(\xi_{d,c}-\xi_{d,m}\right)}^{2}}{\xi_{d,c}{}^{2}},$$

$$SLDE=\sum_{i=1}^{n}{\left(log\left|\xi_{d,c}\right|-log\left|\xi_{d,m}\right|\right)}^{2},$$

$$RMSE=\sqrt{\frac{1}{n}\sum_{i=1}^{n}{\left(\xi_{d,c}-\xi_{d,m}\right)}^{2}},$$

where *n* indicates the total number of data, and *ξ_d,c_* and *ξ_d,m_* indicate the computed and observed velocity-dip-positions, respectively. The combined use of all four errors allows providing a more accurate and complete evaluation, giving more information on the goodness-of-fit of the model. In particular, the scale-independency and interpretability advantages of the average percentage relative error (APRE) allow measuring the model accuracy but produce either infinite or undefined values for zero or observed values close to zero. The sum of squared relative error (SSRE) works similarly, although it is more sensitive to larger relative errors than to smaller ones, while the sum of logarithmic deviation error (SLDE) emphasizes low-magnitude errors. Finally, the root mean square error (RMSE) takes into account error distribution and is very sensitive to outliers.

Table 2 shows how the proposed model is characterized by low errors and great accuracy, thus giving a good representation of the experimental measurements. This demonstrates the model ability to determine the velocity-dip both in narrow channels, where the maximum velocity is below the free surface due to the presence of secondary flows generated by sidewalls, and in wide channels, where the dip-phenomenon is less evident. In addition, the model is applicable over the entire cross-section and for a large range of flow conditions and smooth/rough boundaries.

## 6. Conclusions

Predicting the velocity profile in open channel flows is usually a complex task in hydraulics because of the presence of anisotropic turbulence tensors, which lead to the formation of secondary currents in the channel cross-sections. These secondary flows cause the maximum velocity to be located below the free surface and the negative gradient of the velocity distribution to be vertical above the maximum velocity location.

A theoretical model has been developed in this paper to calculate the velocity-dip-position based on the principle of Shannon’s maximum information entropy. This new model only requires the knowledge of the entropic parameter, which has results that are constant in a given cross-section with varying discharge and might have the same value in cross-sections having the same morphological characteristics.

The general applicability of the model is confirmed by experimental coefficients obtained from a wide set of data referring to channels with various characteristics, such as alignment, slope, roughness, geometric shape, and hence velocity and shear stress distributions.

Four different errors were calculated for all the literature data to prove the accuracy of this law, whose results show how it fits very well with the observed velocity-dip-positions for both narrow and wide open channels, including steady and unsteady flows, thus overcoming the limits of most of the existing models.

## Figures and Tables

**Table 1:** Geometric and kinematic characteristics of experimental data.

Data Sets	*Q* (m^3^/s)	*A_r_*	*U_m_* (m/s)	*u_max_* (m/s)	*ξ_d_ = Y_d_/D*
Kironoto and Graf (1994)	0.022–0.081	2.07–6.90	0.34–0.50	0.40–0.58	0.78–1
Ardiclioglu et al. (2005)	0.007–0.037	4.24–12.01	0.19–0.63	0.25–0.80	0.71–1
Greco and Mirauda (2015)	0.007–0.076	2.22–7.57	0.19–0.76	0.34–1.08	0.52–0.90
Einstein and Chien (1955)	0.074–0.085	1.70–2.55	1.36–2.04	1.58–2.30	0.29–0.63
Guy et al. (1966)	0.05–0.64	7.00–29.00	0.21–1.85	0.25–2.34	0.51–1
Lyn (1987)	0.009–0.013	4.05–4.70	0.63–0.87	0.75–1.02	0.80–0.96
Coleman (1986)	0.064	2.04–2.13	0.93–0.99	1.03–1.12	0.71–0.89
Valiani (1988)	0.023–0.024	3.63–3.83	0.63–0.66	0.69–0.74	0.55–0.89
Graf and Cellino (2002)	0.049–0.065	5.00	0.68–0.92	0.79–1.08	0.81–0.95
Moramarco et al. (2004)	96.53–541.58	3.2–3.4	1.08–2.12	2.02–3.36	0.55–0.99
Greco (2015)	0.07–9.31	9.32–96.15	0.14–2.71	0.21–3.87	0.59–0.91
Mirauda et al. (2018)	0.5–197.9	8.0–92.5	0.09–1.83	0.13–2.54	0.59–0.95

*Q* = water discharge; *A_r_* = aspect ratio; *U_m_* = mean (depth averaged) velocity; *u_max_* = maximum velocity; *ξ_d_* = dimensionless dip-position; *Y_d_* = location of *u_max_* from bed; *D* = water depth.

**Table 2:** Errors estimation for the tested dip-position law.

*Error Indicators*	*Value*
Average percentage relative error (APRE)	7.43
Sum of squared relative error (SSRE)	0.60
Sum of logarithmic deviation error (SLDE)	0.14
Root mean square (RMSE)	0.14
